# Controversies in Surgical Staging of Endometrial Cancer

**DOI:** 10.1155/2010/181963

**Published:** 2010-06-23

**Authors:** R. Seracchioli, S. Solfrini, M. Mabrouk, C. Facchini, N. Di Donato, L. Manuzzi, L. Savelli, S. Venturoli

**Affiliations:** Minimally Invasive Gynaecological Surgery Unit, S. Orsola-Malpighi Hospital, University of Bologna, 40138 Bologna, Italy

## Abstract

Endometrial cancer is the most common gynaecological malignancy and its incidence is increasing. In 1998, international federation of gynaecologists and obstetricians (FIGO) required a change from clinical to surgical staging in endometrial cancer, introducing pelvic and paraaortic lymphadenectomy. This staging requirement raised controversies around the importance of determining nodal status and impact of lymphadenectomy on outcomes. There is agreement about the prognostic value of lymphadenectomy, but its extent, therapeutic value, and benefits in terms of survival are still matter of debate, especially in early stages. Accurate preoperative risk stratification can guide to the appropriate type of surgery by selecting patients who benefit of lymphadenectomy. However, available preoperative and intraoperative investigations are not highly accurate methods to detect lymph nodes and a complete surgical staging remains the most precise method to evaluate extrauterine spread of the disease. Laparotomy has always been considered the standard approach for endometrial cancer surgical staging. Traditional and robotic-assisted laparoscopic techniques seem to provide equivalent results in terms of disease-free survival and overall survival compared to laparotomy. These minimally invasive approaches demonstrated additional benefits as shorter hospital stay, less use of pain killers, lower rate of complications and improved quality of life.

## 1. Introduction

Endometrial cancer is one of the most common gynaecological malignancies in developed countries and, unfortunately, the incidence of endometrial cancer is rising. This may be attributed to risk factors, like increased life expectancy, obesity, diabetes, late menopause, and use of Tamoxifen [1–3]. Endometrial cancer spreads towards myometrial wall, cervix, and lymphatic stations of pelvic and paraaortic lymph nodes [4]. Prognosis of this malignancy depends on various factors: histological type of the tumour, the depth of invasion into the myometrium, and lymph node involvement [1–4]. 

Surgical management of endometrial cancer is a challenge. It is important to balance risks and benefits of each surgical option, avoiding both over- and undertreatment.

The Gynaecologic Oncology Group (GOG) trial published in 1987, lead to a crucial change from clinical to surgical staging. By this study, pelvic and paraaortic lymphadenectomy have been introduced in the oncological practice of endometrial cancer on the basis of the international federation of gynaecologists and obstetricians (FIGO) criteria [5]. The new FIGO classification addressed new information about prognostic predictors. However, the extent of surgical staging, the definition of high-risk patients who benefit from complete staging, numbers of lymph nodes, and anatomical limits in paraaortic area still lack standardisation [6–10].

In the present manuscript, we sought to review the available evidences and to discuss controversies in surgical management of endometrial cancer, considering the following items:

1. Complete surgical staging: Role of lymphadenectomy in endometrial cancer;
2. Preoperative evaluation: Predictors of lymph node metastasis;
3. Intraoperative detection of lymph node metastasis;
4. Extent of lymphadenectomy;
5. Surgical approach for staging of endometrial cancer.

## 2. Complete Surgical Staging: Role of Lymphadenectomy in Endometrial Cancer

A complete surgical staging, including lymphadenectomy is the gold standard to evaluate lymph node involvement, the most common site of extrauterine spread of endometrial cancer. However, the exact role, indications, and extent of lymphadenectomy in endometrial cancer patients remain controversial [4, 6, 11–13]. A recent Cochrane protocol confirmed the prognostic role of lymphadenectomy, while its therapeutic role, the advantages in terms of survival, and extent of anatomical standardization are under debate [4]. Lymph node metastasis has been described, as well as one of the strongest predictors of disease recurrence and as a guide for subsequent adjuvant therapy in patients with positive lymph nodes. Patients with stage I disease have more than 90% 5-year survival rate compared to those with nodal metastasis who have survival rates ranging from 38% to 75% [14]. 

In a retrospective study, Bernardini et al. verified that a substantial number of patients with grade 1 endometrial cancer, based on preoperative and intra operative assessment, had higher grade disease on final pathology. Lymphadenectomy did not affect survival in these patients; however, it could identify patients with advanced disease and assist in tailoring adjuvant therapy for those with adverse risk factors [15]. 

Nevertheless, the exact therapeutic benefit in terms of survival associated with lymphadenectomy is difficult to define, especially in early stages. 

Recently, a multicentric randomized controlled trial (ASTEC) demonstrated no evidence of benefit for systematic lymphadenectomy in terms of overall, disease-specific, and recurrence-free survival in women with endometrial cancer. A total of 1408 women, with a preoperative diagnosis of endometrial cancer confined to the corpus uteri were randomized to standard surgery or standard surgery plus pelvic lymphadenectomy. A similar proportion of women in both groups received postoperative radiotherapy. After a median follow up of 37 months, there was no difference in overall survival between two groups and the analysis of disease or treatment-related death was in favour of the standard surgery group. Moreover, there was a significant benefit in recurrence-free survival for the standard surgery group, and surgical complication rates were higher in the lymphadenectomy group [16]. The results of the ASTEC trial, however, have been widely discussed. One important concern to limit the generalization of these results is the low number of lymph nodes (median of 12 lymph nodes) removed in the lymphadenectomy group. In the literature, excision of higher number of nodes was proved to have an effect on overall survival, especially in patients with high-risk and intermediate-risk endometrial cancer [13, 17–19]. In addition, the ASTEC study did not assess the paraaortic nodes, which can be involved in up to 67% of patients with pelvic lymph node metastasis as demonstrated by Mariani et al. [20]. Another issue to be considered is the high rate of low-risk patients (STAGE 1A-1B grade 1-2) included in lymphadenectomy group, and subsequently low rates of pelvic node metastasis. Finally, the follow up duration was considered too short for a survival study of a malignant disease. 

Furthermore, complete staging was not found to improve overall survival and disease-free survival in another RCT that compared treatment of early stage endometrial carcinoma with and without systematic pelvic lymphadenectomy [21].

A retrospective database review considered 12,333 patients undergoing surgical staging by lymphadenectomy and stratified them in groups: a low-risk (Stage IA, all grades and Stage IB, grade 1 and 2) and a medium- to high-risk group (Stage IB, grade 3 and Stage IC-IV, all grades). In low-risk group, there was no significant benefit of nodal resection, while a multivariate analysis demonstrated that in the medium- to high-risk group a more extended nodal resection was associated with increased 5-year survival [13].

## 3. Preoperative Evaluation: Predictors of Lymph Node Metastasis

There is general agreement that definitive staging of endometrial cancer is based on pathological examination, but an accurate preoperative risk stratification guides to the appropriate type of surgery, avoiding the morbidity associated with unnecessary procedures [2, 6].

Both histopathological and clinical risk factors have been identified as predictors of lymph node involvement: histological type, grade of tumour, myometrial invasion, and cervical infiltration [2–6, 14, 22, 23].

### 3.1. Preoperative Endometrial Biopsy

Tumour histological grading remains the most important preoperative factor in identifying risk status. There is only poor correlation between histological grade of tumour based on endometrial biopsy or D&C and final pathology. Histological upgrading was demonstrated in 18% of endometrial cancer patients after definitive histological examination [2, 24, 25]. On the other hand, the identification of clear cell or papillary serous carcinoma was demonstrated to have increased risk of distant metastasis, even in case of endometrial confined lesions [26].

### 3.2. Imaging Modalities and Risk Stratification

As regards preoperative clinical staging, several studies proposed that identification of patients with deep myometrial invasions (more than 50%; FIGO stage IC) and preoperative knowledge of cervical stroma infiltration (FIGO stage IIb) are important determinants for surgical decision [27, 28]. Several techniques are used to evaluate the depth of myometrial invasion and cervical infiltration. In this context, Magnetic Resonance Imaging (MRI), Computed Tomography (CT), and Transvaginal Sonography (TVS) are the main diagnostic tools used. Comparing the diagnostic accuracy of these techniques, several studies demonstrated no significant differences in performance for both myometrial extent and cervical invasion [29, 30]. A recent prospective study compared the high-frequency (5.0–9.0 MHz) TVS and contrast-enhanced MRI in preoperative staging of endometrial cancer. Authors concluded that, in expert hands, TVS seems to be a feasible and more economic imaging method with accuracy comparable with MRI and it can be proposed as first-line option for evaluation of myometrial invasions and cervical spread [31]. 

Some imaging modalities can also investigate the status of lymph nodes, but the results, to date, have been disappointing. MRI and CT/PET are statistically comparable, but have only a limited specificity in detecting pelvic and paraaortic node metastasis [29, 30].

## 4. Intraoperative Detection of Lymph Node Metastasis

There is currently no validated method for predicting lymph node metastasis. Accordingly, many authors support a comprehensive surgical staging for endometrial cancer. Although intraoperative evaluation methods for lymph node metastasis, as frozen section examination and node palpations, are often used in surgical oncological practice, there is scientific agreement that they are inaccurate. 

### 4.1. Lymph Node Palpation

Girardi et al. found that 37% of nodal metastases were less than 2 mm and only 7% larger than 2 cm [32]. Several authors demonstrated the high false-negative rates for intraoperative lymph node palpation (26% by Eltabbakh and 36% by Arango et al.) [33, 34].

### 4.2. Frozen Section

The incidence of lymph node metastasis is related to the depth of invasion and tumor grade. Intraoperative frozen section might identify patients who are at risk for extrauterine spread and required complete surgical staging. 

Frozen section may help to further stratify for the risk of final pathology but is not entirely accurate [35]. To date, available evidence does not clearly support modulating the extent of surgical staging according to the results of frozen section examination.

Case et al. evaluated in a prospective-blinded study the accuracy of frozen section in surgical management of endometrial cancer. There was a poor correlation between frozen and final section: only 67% for invasion depth and 58% for tumour grade. This study demonstrated a clinically relevant upstaging in 18% of patient who underwent lymphadenectomy [36]. Another study by Frumovitz et al. verified that the combination of intraoperative frozen section analysis for histological grade and depth of myometrial invasion correlates poorly with final pathologic grade and stage in patients with apparent grade I and II tumor [37]. 

The finding of negative pelvic nodes at intraoperative frozen section has been proposed to guide further surgical management during surgical staging of endometrial cancer. A recent study by Papadia et al. confirmed that frozen section underestimated the risk of lymph node involvement in 16% of cases when compared with final section pathology [38]. Another trial by Pristauz et al. verified that intraoperative frozen section of pelvic nodes is not accurate to tailor the extent of lymphadenectomy. In this study, examination of pelvic nodes had a sensitivity of 41% and a false negative rate of 59% [39].

### 4.3. Sentinel Lymph Node Examination

In an effort to decrease the morbidity resulting from lymphadenectomy, several authors proposed the sentinel lymph node (SN) detection approach. Although it is still under investigation, this technique has several potential benefits in surgical management of endometrial cancer. Data on feasibility and utility are rapidly increasing. However, few studies have concluded the feasibility of SN in endometrial cancer [40–50]. It has been verified by many authors that SN detection may help to evaluate regional lymphatic status and to select the group of patients that must be submitted to a complete lymphadenectomy, avoiding surgical invasiveness in early stage cancer [49, 50]. Most investigators performed intramyometrial or intracervical punctures [40–50]. The identification rates were 61.5% to 67%, when blue dye was injected into the subserosal myometrium of the fundus, and 83% by additional injections of blue dye into the cervix [40]. The modern trend in lymphatic mapping for endometrial cancer is through subendometrial hysteroscopic injection. Delaloye et al. published a study evaluating hysteroscopic injection of patent blue dye and radioactive tracer beneath the tumour of 60 patients with endometrial carcinoma, sentinel nodes were identified in 49 of 60 patients (82%) [50].

## 5. Extent of Lymphadenectomy

Actually, the extent and anatomical limits of lymphadenectomy in endometrial cancer is another topic of scientific debate. The GOG (Gynaecologic Oncology Group) has standardized the surgical limits of pelvic and paraaortic lymphadenectomy including the genitofemoral nerve laterally, the hypogastric artery medially, the obturator nerve posteriorly, the circumflex iliac vein caudally, and inferior mesenteric artery (IMA) as cranial limit when performing paraaortic lymphadenectomy [51]. 

### 5.1. Paraaortic Lymphadenectomy: To Do or Not To Do?

Retroperitoneal lymph node metastasis is a significant prognostic factor for patients with endometrial cancer. The risk of paraaortic nodal metastasis can be related to the presence of adnexal metastasis and/or pelvic lymph nodes metastasis: paraaortic lymph nodes are positive in 38%–52% of cases with positive pelvic lymph nodes, in 20%–57% with adnexal metastasis, and in only 2% with negative pelvic nodes [52]. In other trials, a range from 28.6% to 66.7% of patients with pelvic metastasis had concomitant positive paraaortic nodes [5, 52–54]. 

Mariani et al. demonstrated that 47% of patients with pelvic lymph nodes metastasis also have positive paraaortic lymph nodes or will submit a relapse in paraaortic region [20]. Furthermore there are reports in literature showing that increasing number of positive pelvic nodes is associated with paraaortic metastasis [55, 56]. Fujimoto et al. reported the therapeutic significance of complete paraaortic lymphadenectomy in 63 patients with stage IIIC endometrial carcinoma. Despite there was no significant difference in disease-related survival, the authors found an improvement in disease-related survival in patients with two or more positive lymph nodes [57]. Mariani et al. reported the potential therapeutic role of paraaortic lymphadenectomy in node positive patient with endometrial cancer [58, 59]. The 5-year progression free and overall survival rates were significantly better in paraaortic lymphadenectomy group. From the available studies we could conclude that paraaortic lymphadenectomy might have a therapeutic role at least for high-risk patients. However, further RCT are needed to confirm this conclusion.

### 5.2. Cranial Limit of Paraaortic Lymphadenectomy: Where to Arrive?

Moreover, the cranial extent of paraaortic lymphadenectomy has recently been a matter of debate. A prospective study by Mariani et al. evaluated patients with high-risk endometrial cancer requiring a complete lymphadenectomy. Considering patients with positive lymph nodes, 77% of them had paraaortic metastasis above the IMA. The authors emphasized the importance of systematic pelvic and paraaortic lymphadenectomy up to the renal vessels with excision of the gonadal veins [20].

### 5.3. Number of Removed Lymph Nodes

Another controversial issue is the number of lymph nodes that must be removed for proper surgical staging. Lutman et al. found that pelvic lymph node count ≥12 is an independent prognostic factor for both overall and progression-free survival in patients with FIGO stage I and II with high-risk histology [18]. Another study by Cragun et al. confirmed that patients with grade 3 endometrial cancers having more than 11 pelvic nodes removed had improved overall survival and progression-free survival compared with patients with 11 or fewer nodes removed [19]. Chan et al. have shown a correlation between the increasing number of lymph nodes removed and number of nodal metastasis. They concluded that the removal of 21 to 25 nodes was considered to significantly increase the probability of detecting at least one lymph node metastasis [60].

## 6. Surgical Approach for Staging of Endometrial Cancer

Surgical treatment of endometrial cancer has traditionally been through laparotomy. Nevertheless, in the last 15 years, the use of minimally invasive techniques is getting widely accepted by many authors [61–64]. The laparoscopic approach can be either laparoscopic-assisted vaginal hysterectomy (LAVH) or total laparoscopic hysterectomy (TLH). These procedures proved feasible and safe when compared with laparotomy [61–69]. 

The primary endpoint for trials comparing laparoscopic and laparotomic approach is to demonstrate the equivalence in terms of staging completeness and survival rates. Other endpoints are hospital stay, postoperative pain, quality of life (QOL), and health costs of the procedures. The randomized study of the Gynaecologic Oncology Group (GOG-LAP2) considered the laparoscopic and laparotomic surgery for stage I-IIa, grade 1–3 endometrial cancer. There were no significant differences in terms of staging completeness, lymph node metastasis, rate of perioperative complications, and mortality between the two procedures. This trial verified that although the laparoscopic approach has a longer operative time, it has the advantage of a shorter hospital stay [61]. The quality of life is another important index in evaluation of the therapeutic role of laparoscopy in endometrial cancer. The same study (GOG-LAP2), through examination of QOL indicators and from the results of validated questionnaire SF-36, demonstrated that laparoscopy has a significant advantage in terms of quality of life within the first 6 weeks. Data from GOG-LAP2 on rate of relapse and long-term survival are not yet available [61]. 

A recent meta-analysis showed that, in early stages, laparoscopic approach is equally effective as laparotomic one in terms of overall survival, disease-free, and cancer-related survival. Both techniques were proven equal in terms of intraoperative complications and number of pelvic and paraaortic node yield. Laparoscopy had additional benefits like lower blood loss and fewer postoperative complications rates; however, it had other disadvantages in terms of longer operative time and learning curve [63]. 

### 6.1. Use of Laparoscopy in Obese Patients with Endometrial Cancer

The feasibility and safety of the use of laparoscopy in obese women with endometrial cancer are other issues of concern. Obesity and comorbidity were considered, for many years, contraindications for laparoscopic approach. However, comparative studies demonstrated that patients with increased surgical risk (obese and elderly) are the ones who most benefit from the minimally invasive approach, in terms of reduction of operative morbidity (e.g., laparotomic wound infections and bowel obstruction), postoperative pain, hospital stay, and time to return to full activity [65–70].

A recent study compared the safety and efficacy of laparoscopy and laparotomy in surgical staging of early stages (FIGO I-II) in obese women. Authors found no significant differences among the two groups regarding mean operative time, with a significantly higher blood loss and hospital stay in patients treated by laparotomy [67].

Another study comparing obese and nonobese women with laparoscopically treated stage I endometrial cancer found no difference in operative time, pelvic node removed, and complications, although obese group had higher blood loss [68].

### 6.2. Actual Use of Laparoscopy in Endometrial Cancer Management

Despite the controversies regarding endometrial cancer staging by laparoscopy, the use of this procedure in oncological practice is increasingly rising. A recent follow-up survey among members of the Society of Gynaecological Oncology found an overall increase in the use and indications for minimally invasive surgery in gynaecological oncology. 91% of responders indicated that they perform laparoscopy in their surgical practice. Laparoscopic hysterectomy for endometrial cancer staging was the most frequent procedure performed (43%) [71].

### 6.3. Robotic-Assisted Surgical Approach

Since 2005, there has been a considerable increase in the published literature describing the use of robotic-assisted surgery for endometrial cancer staging [70–84]. When compared to laparotomy, the robotic-assisted surgery had significantly longer mean operative time, but lymph node yields were comparable to the open surgery. The length of hospital stay, blood loss, and postoperative complication rates were significantly lower for robotically operated patients [79, 80]. 

Boggess et al. compared three surgical methods for endometrial cancer staging: laparotomy, laparoscopy, and robotic-assisted approach. Patients operated by robotic technique had shortest hospital stay, lowest estimated blood loss, and highest lymph node yield. Operative time was the longest for laparoscopy followed by robotics, with a similar laparotomic conversion rates for robotic and laparoscopic groups [81]. Robotic-assisted approach has been also proposed as a good and feasible option for comprehensive surgical staging in obese women with endometrial cancer [82]. Moreover, this technique may have particular advantages for both the obese and morbidly obese patients affected with endometrial cancer, when compared to laparoscopic approach [83, 84].

## 7. Conclusions

Surgical staging for endometrial cancer represents certain benefits: firstly, it is the gold standard to assess the disease extent. Secondly, it also has a prognostic role and guides for further treatment. The therapeutic value of lymphadenectomy has not been proven in prospective studies, especially in low-risk cases at preoperative staging. 

There are many predictors of lymph nodes involvement useful to evaluate patient's risk categories and to guide surgical management. TVS and MRI may accurately detect the depth of myometrial invasion and cervical spread of the disease, but preoperative imaging cannot accurately assess lymph node involvement. Intraoperative assessment of node involvement and myometrial invasion does not have the sensitivity and specificity to select women who can avoid lymphadenectomy from the surgical procedure.

A great challenge in surgical management of endometrial cancer is standardisation of pelvic and paraaortic lymphadenectomy strategies, in order to avoid unnecessary procedures and to offer complete staging with high survival rates. 

The morbidity of lymphadenectomy appears to be reduced with the use of laparoscopy. Numerous trials have demonstrated the safety and feasibility of laparoscopy in complete surgical staging for early stages of endometrial cancer with similar nodes yields, recurrence and survival outcomes. As expected, significant improvements in early and late postoperative complications, a shorter hospital stay, a better quality of life, and less overall treatment costs were demonstrated in many comparative studies between laparotomy and laparoscopy. Laparoscopic approach is safe and feasible also in obese and elderly woman with early stage endometrial cancer, with low rate of conversion, shorter hospital stay, and a faster return to full activity compared with laparotomy.

The role of robotics in endometrial cancer staging continues to evolve and has yet to be determined definitively. Most studies about robotic surgery show that it is a feasible and safe option, especially for obese patients.
